# A Mixture Modeling Framework for Differential Analysis of High-Throughput Data

**DOI:** 10.1155/2014/758718

**Published:** 2014-06-25

**Authors:** Cenny Taslim, Shili Lin

**Affiliations:** Department of Statistics, The Ohio State University, Columbus, OH 43210, USA

## Abstract

The inventions of microarray and next generation sequencing technologies have revolutionized research in genomics; platforms have led to massive amount of data in gene expression, methylation, and protein-DNA interactions. A common theme among a number of biological problems using high-throughput technologies is differential analysis. Despite the common theme, different data types have their own unique features, creating a “moving target” scenario. As such, methods specifically designed for one data type may not lead to satisfactory results when applied to another data type. To meet this challenge so that not only currently existing data types but also data from future problems, platforms, or experiments can be analyzed, we propose a mixture modeling framework that is flexible enough to automatically adapt to any moving target. More specifically, the approach considers several classes of mixture models and essentially provides a model-based procedure whose model is adaptive to the particular data being analyzed. We demonstrate the utility of the methodology by applying it to three types of real data: gene expression, methylation, and ChIP-seq. We also carried out simulations to gauge the performance and showed that the approach can be more efficient than any individual model without inflating type I error.

## 1. Introduction

With the completion of the human genome project more than a decade ago, large-scale approaches to biological research are advancing rapidly. In particular, the inventions of microarray and next generation sequencing technologies have revolutionized research in genomics; such high-throughput platforms have led to massive amount of data. Depending on the study, each type of experiment generates data with different characteristics. Among them are cDNA microarrays or RNA-seq for measuring changes in expression levels of thousands of genes simultaneously [1, 2]; ChIP-chip tiling arrays or ChIP-seq for studying genome-wide protein-DNA interactions [3, 4]; and differential methylation hybridization microarrays or whole genome bisulfite sequencing for performing whole genome DNA methylation profiling study [5, 6]. A common theme of interest for biologists when they employ these experiments is to perform differential analysis [7–12]. For example, in gene expression profiling, be it microarray or sequencing based, there is an interest in finding genes that are differentially expressed. For epigenetic profiling of cancer samples, it is of interest to find CpG islands that are differentially methylated between cancerous and normal cells. On the other hand, ChIP-seq data are frequently used to interrogate protein binding differentiation under two different conditions. Over the past decade, methods have been proposed for each type of data when new platforms/technologies were launched. Despite the common theme, different data types have their own unique features, creating a “moving target” scenario. As such, methods specifically designed for one data type may not lead to satisfactory results when applied to another data type. Furthermore, new data types from new biological experiments will continue to emerge as we are entering a new era of discovery [13, 14]. As such, it would be desirable to have a unified approach that would provide satisfactory solutions to multitype data, both those currently available and those that will become available in the future. To meet this challenge so that not only currently existing data types but also data from future problems, platforms, or experiments can be analyzed, we propose a mixture modeling framework that is flexible enough to automatically adapt to any moving target. That is, the model we are proposing is adaptive to the data being analyzed rather than being fixed. More specifically, the approach considers several classes of mixture models and essentially provides a model-based procedure with the following features: (1) use of an ensemble of multiclass models, (2) models within each class adapting to the data being analyzed, and (3) flexible scheme for component classification. Thus, depending on the underlying distribution of the data being analyzed, the model will adapt accordingly to provide the best fit, which, as we demonstrate through simulation, can lead to improved power and sensitivity of differential identification without inflating type I error. To illustrate the utility of the method, we employ it to analyze three diverse types of high-throughput data, each of which has led to improved fit compared to a single-model analysis.

## 2. Materials and Methods

### 2.1. Synopsis of the Ensemble Approach

Mixture model-based approaches have been proposed specifically for different data types. Here, we propose an approach that tries to synthesize the advantages of these approaches into one single package. Depending on the data being analyzed, this ensemble approach will select the model that best fits the data and perform model-based classification. The first mixture model being considered for the ensemble is the gamma-normal-gamma (GNG) model proposed for analyzing DNA methylation data [15]. It uses a special case of the gamma distribution (exponential) to capture data coming from differential group and utilizes multiple normal components to capture the nondifferentiating methylated group allowing for small biases even after normalization. We integrate this model with uniform-normal mixture model (NUDGE) proposed by Dean and Raftery [16] which uses one uniform and one normal component to analyze gene expression data. To extend the applicability of the ensemble approach to other omic data types, we add to this ensemble an extension of NUDGE, which we call iNUDGE, to improve the fit by following the idea from GNG using multiple normal components. A robust weighting scheme for GNG was also extended to (i)NUDGE. In addition, we allow some of the normal components to be classified as capturing differentiated observations based on their locations and scale parameters, further increasing the flexibility of the ensemble model. We note that this feature differs from the intended use of the normal component(s) in GNG and NUDGE. Depending on the underlying distribution of the data, the best overall model among the three classes will be selected and used for inferences. The ensemble nature of this procedure makes it highly adaptable to data from various platforms. We demonstrate this capability by applying it to three types of data: gene expression, DNA methylation, and ChIP-seq. In what follows, we describe our ensemble model, parameters estimation, model selection, and finally model-based classification.

### 2.2. Ensemble of Finite Mixture Models

In the proposed ensemble approach, we integrate advantages from different models by considering multiple underlying distributions. Specifically, a collection of three classes of mixture models are utilized. Each class of models is designed to fit the normalized data that are usually expressed as (log) differences under two experimental conditions, for example, healthy versus diseased or before versus after treatment.

Let *f*(*y*) be the unknown density function of the normalized data point *y*, which is modeled as

(1) $$\begin{array}{c} f\left(y;\Psi \right)=\left(1-\pi \right)f_{0}\left(y;\Psi_{0}\right)+\pi f_{1}\left(y;\Psi_{1}\right), \end{array}$$

where Ψ, Ψ_0_, and Ψ_1_ are the underlying model parameters for the mixture and each of the two components, respectively, and will be specified as the formulation enfolds. In this first level of mixture, *f*_1_ is designated to capture differential elements (overdispersion) whereas *f*_0_ is for those that are more centrally located. Nevertheless, *f*_0_ may also be used to identify differential observations, as detailed in the second level of mixture modeling. Specifically, we model *f*_1_ and *f*_0_ as follows:

(2) $$\begin{array}{c} f_{1}\left(y;\Psi_{1}\right)=\{\begin{array}{cc} U_{\left[a,b\right]}\left(y\right) & \text{for}\;\left(\text{i}\right)\text{NUDGE}\\ \rho E_{1}\left(-y\times I\left\{y<-\xi_{1}\right\};\beta_{1}\right) & \\ \;+\left(1-\rho \right) & \\ \;\times E_{2}\left(y\times I\left\{y>\xi_{2}\right\};\beta_{2}\right) & \text{for}\;\text{GNG}, \end{array}\\ f_{0}\left(y;\Psi_{0}\right)=\{\begin{array}{cc} N\left(y;\mu ,\sigma^{2}\right) & \text{for}\;\text{NUDGE}\\ \overset{K}{\underset{k=1}{\sum }}\gamma_{k} & \\ \;\times N\left(y;\mu_{k},\sigma_{k}^{2}\right), & \\ \;\overset{K}{\underset{k=1}{\sum }}\gamma_{k}=1 & \text{for}\;\text{iNUDGE}\;\text{and}\;\text{GNG}. \end{array} \end{array}$$

As we can see from the above modeling, the overdispersion in the data is captured by either a uniform distribution or a mixture of two exponential distributions (special case of gamma). The parameters of the uniform distributions, *a* and *b*, are part of the model parameters (i.e., *a*, *b* ∈ Ψ_1_) and so are the scale parameters and the mixing proportion of the exponential distributions (i.e., *ρ*, *β*_1_, *β*_2_ ∈ Ψ_1_). The location parameters, *ξ*_1_ and *ξ*_2_ both >0, are assumed to be known. In practice, ${\hat{\xi }}_{1}=|\max(y<0)|$ and ${\hat{\xi }}_{2}=|\min(y>0)|$ may be used as estimates of *ξ*_1_ and *ξ*_2_. The more centrally located data are represented by either a single normal distribution or a mixture of normal distributions. The location and scale parameters are part of the model parameters, that is, *μ*, *σ* ^2^, *μ*_*k*_, *σ*_*k*_^2^ ∈ Ψ_0_, and so are the mixing proportions *γ*_*k*_ and the number of components in the mixture, *K*; that is *γ*_*k*_, *K* ∈ Ψ_0_. Thus, Ψ = {*π*} ∪ Ψ_0_ ∪ Ψ_1_. Finally, *I*{·} is the usual indicator function that is equal to 1 if the condition in { } is satisfied; otherwise, it is 0. Since any distribution can be well represented by a mixture of normal distributions, both *f*_1_ and some components of the normal mixture will be designated as “differential” components, as detailed below.

### 2.3. Robust Parameter Estimation

In order to get a robust estimation of model parameters, following GNG, we use a weighted likelihood function in our ensemble model:

(3) $$\begin{array}{c} l\left(\Psi \right)=\overset{n}{\underset{i=1}{\sum }}w_{i}\log f\left(y_{i};\Psi \right), \end{array}$$

where *y*_*i*_, for *i* = 1,2,…, *n*, are the normalized observed data and 0 ≤ *w*_*i*_ ≤ 1 are some prespecified weights.

Weighted likelihood is used because we want to downgrade the contributions from observations with small “intensities.” For example, in modeling log-ratio, we want to distinguish data points with the same log-ratio but vastly different magnitudes in their individual intensities. If we let *u* be the average log intensities (standardized to mean zero and standard deviation 1), then the lower half Huber's weight function:

(4) $$\begin{array}{c} w\left(u\right)=\{\begin{array}{cc} 1, & \text{if}\;u>-c\\ \frac{c}{\left|u\right|} & \text{if}\;u\leq -c, \end{array} \end{array}$$

where *c* = 1.345, can be used to downweigh those with smaller average intensities. In addition to Huber's weight function, Tukey's bisquare function may also be used [17]. Further, an upper half or a two-tailed weight function can be used if justifiable for a particular data type or study goal.

The EM algorithm is used to fit each class of models under the ensemble. The stopping criteria for our EM algorithm are when either ||Ψ_(*m*+1)_ − Ψ_(*m*)_|| < *ϵ* or a maximum number of iterations *M* are reached. In our simulation and data analysis, we set *ϵ* = 10^−5^ and *M* = 2000, which are also the default setting in the program implementing the ensemble approach.

### 2.4. Model Selection and Model-Based Classification

In both GNG and iNUDGE models, we first need to determine *K*, the number of normal components in the model, also known as the order of the model. In our analysis, we examine models with *K* = 1,2,… and choose *K* that maximizes the Bayesian information criterion (BIC [18]). We use BIC as it is in favor of parsimonious model since the penalty for additional parameters is stronger than the Akaike information criterion (AIC [19]). That is, when selecting the order of the model, we want to be extra careful not to choose models that are too complex. After identifying the best model within each class, we use the AIC to select the overall best model among the three classes. The use of this balanced model selection approach is not only to prevent the selection of a model that is too complex (thus using BIC within each class) but, in the meantime, also to avoid choosing a model that is overly simple (thus using AIC when selecting among the classes).

Using the best model selected, a two-step approach is taken to classify each observation as differential or not. In the first step, we classify a normal component *N*(*μ*_*k*_, *σ*_*k*_^2^) as a differential one if one of its tails captures observations that are “outliers” in the overall distribution:

(5) $$\begin{array}{c} \left|\mu_{k}\right|+2\times \sigma_{k}>1.5\times \text{IQR}, \end{array}$$

where IQR is the interquartile range of the entire dataset. The normal components that are not labeled as differential are called “nondifferential.”

After each normal component is labeled, we compute the local false discovery rate (fdr) proposed by Efron [20] and adapted by Khalili et al. [15] for each observation:

(6) $$\begin{array}{c} \text{fdr}\left(y_{i}\right)=\frac{f_{\text{nd}}\left(y_{i},{\hat{\Psi }}_{0}\right)}{f\left(y_{i};\hat{\Psi }\right)},\;\;\;\;\;\;\;\forall i\in n, \end{array}$$

where *f*_nd_ is composed of normal components that are designated as nondifferential. We then classify observation *y*_*i*_ with weight *w*_*i*_ to be a differential element if fdr(*y*_*i*_)/*w*_*i*_ ≤ *z*_0_, for some threshold value *z*_0_.

### 2.5. Software

The method presented in this paper has been implemented in an R package called DIME (differential identification using mixture ensemble) and is available at http://www.stat.osu.edu/~statgen/SOFTWARE/DIME/ or http://cran.r-project.org/web/packages/DIME/index.html (CRAN).

## 3. Results and Discussion

### 3.1. Simulation Study

Our simulation was modeled after the APO AI gene expression data [21]. Let *x*_*ij*_ be the logarithm of expression level corresponding to the *i*th gene (observation unit) in the *j*th sample (*j* = 1 if it is a control sample and *j* = 2 if it is a treatment sample). For nondifferential genes, we generated *x*_*i*2_ − *x*_*i*1_ by sampling randomly from genes in the APO AI dataset for which the log-ratio is at most one. For differential genes, we simulated the log of expression level in the control sample from a uniform distribution (i.e., *x*_*i*1_ ~ unif (15,30)); we set the log expression level in the treatment sample to be *x*_*i*2_ = *x*_*i*1_ + *Z*_*i*2_ + (2 × *B*_*i*2_ − 1) × *G*_*i*2_, where *Z*_*i*2_ ~ *N*(0,0.7 − 0.02 × *x*_*i*1_), *B*_*i*2_ ~ Bern (0.5), and *G*_*i*2_ followed one of the following three distributions:

(7) $$\begin{array}{c} G_{i2}\sim \{\begin{array}{cc} \left(1\right) & \text{exponential}\left(\beta =1.4286\right)+1,\\ \left(2\right) & \text{uniform}\left(1,4\right),\\ \left(3\right) & \text{normal}\left(\mu =2.5,\sigma =0.75\right). \end{array} \end{array}$$

**Figure 1:**
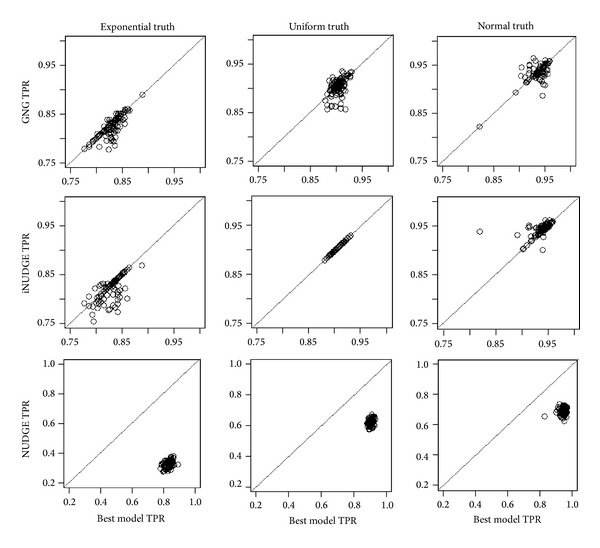
Simulation results comparing the performance of the ensemble approach with each of the three individual classes of models, GNG (row 1), iNUDGE (row 2), and NUDGE (row 3), under 3 different underlying models, exponential (column 1), uniform (column 2), and normal (column 3), representing three different data types.

Note that *Z*_*i*2_ was set such that genes with smaller expression will have larger variance, while *B*_*i*2_ controls over- or underexpression of genes. Further, *G*_*i*2_ represents three different underlying distributions for differential observations to study the performance of the ensemble model under diverse data types. We generated 10,000 genes for which 10% (1000) are differential elements. A total of 100 datasets were simulated under each of the three simulation settings (the three *G* distributions in (7)). In each replicate, we calculated false positive rate (FPR) and true positive rate (TPR) for classifying each gene as differential or nondifferential. Here, TPR is the rate of correct classification of differential genes and FPR is the rate of classifying a gene to be differential when it is in fact a nondifferential gene.  Figure 1 shows the result of the ensemble approach fitting these three types of simulated data. In column 1 (datasets with exponential distribution for *G*), we can see that the best model selected was GNG for a majority of the replicates, which is evident from the fact that the majority of the points are on the diagonal line for the best model versus GNG plot (row 1, column 1). Although iNUDGE also has similar TPR (row 2, column 1), it is more variable (more scattering) with a slightly lower average TPR. In column 2 (datasets with uniform distribution), iNUDGE was chosen as the best model in all replicates, while GNG has slightly lower TPR and more scattering (row 1, column 2), opposite of column 1. For model with underlying normal truth (3rd column), iNUDGE was selected to be the best in some cases whereas in other cases GNG was selected as the best overall model. Overall, regardless of the underlying distribution, the best model selected has comparable or better TPR compared to individual iNUDGE or GNG models. On the other hand, results from NUDGE are associated with a much lower TPR due to its limitation of using only one normal component (row 3). The false positive rates are not shown as they are zero in all replicates.

### 3.2. Real Data Analysis

After confirming the utility of the ensemble approach for handling multiple data types through a simulation study, we analyzed real data sets from ChIP-seq, DNA methylation, and gene expressions. ChIP-seq experiment has become the most commonly used technique to interrogate genome-wide protein-DNA interaction locations in recent years. It has enabled scientists to study transcription factor binding sites with better accuracy and less cost compared to older technology such as ChIP-chip [4]. Such data may be used to capture differential transcription factor binding sites in normal versus cancer samples, which may provide insights as to how cancer-related genes are turned on/off. For more information about ChIP-seq datasets and the methods used in preprocessing, including normalization, see Taslim et al. [11]. DNA methylation is an important factor in heritable epigenetic regulation that has been shown to alter gene expression without changes in DNA sequence. DNA methylation has been associated with many important processes such as genomic imprinting and carcinogenesis [22, 23]. Likewise, differential analysis of gene expression has enabled researchers to find cancer-associated genes and other diseases [24, 25]. For more information on DNA methylation and gene expression datasets and normalization methods, see Khalili et al. [15] and Dean and Raftery [16].  Table 1 provides a summary of the three types of real data used in this section.

#### 3.2.1. ChIP-Seq Data

**Figure 2:**
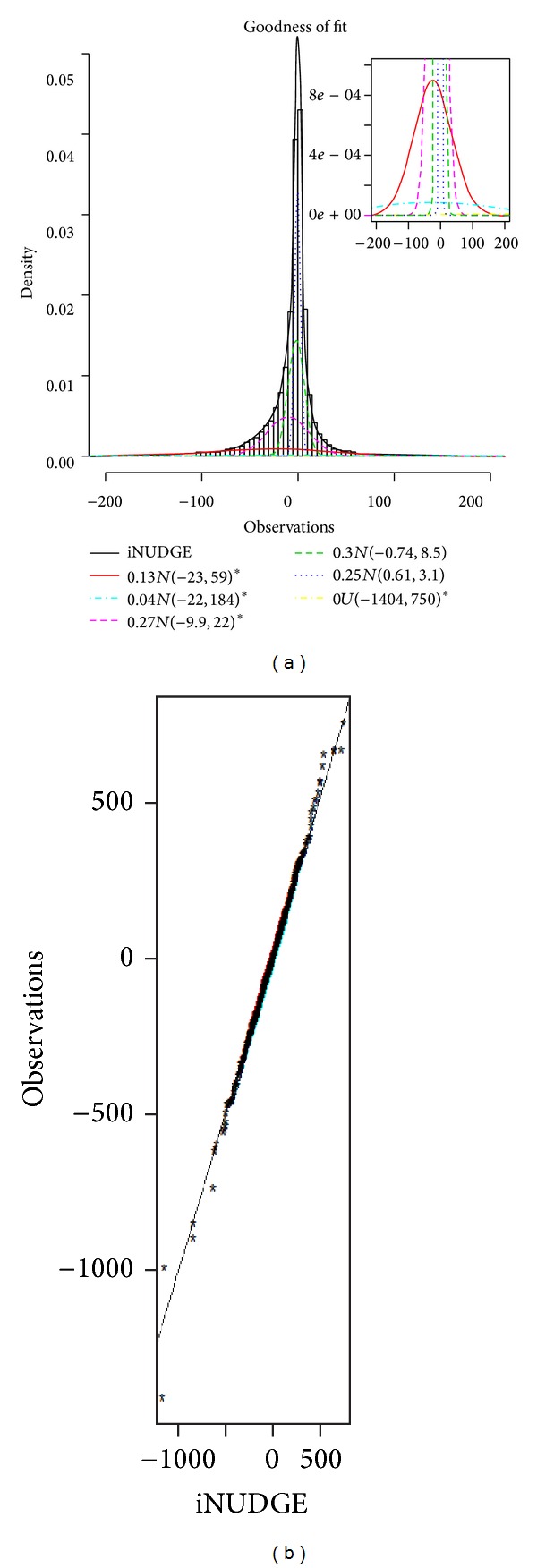
Results from fitting DIME to the ChIP-seq data, where ∗ designates a differential component. (a) The histogram of the methylation data is superimposed by the fitted best model and the individual components (inset: zoomed in view showing the individual components of the fitted model). (b) The QQ-plot of the best model versus the observed normalized ChIP-seq data.

The ensemble model was applied to identify genes associated with enriched polymerase II (Pol II) binding quantity in OHT (tamoxifen resistant breast cancer cell line) compared to normal breast cancer (MCF7). We used the normalized data described in Taslim et al. [11]. The ensemble modeling approach selected iNUDGE as the best overall model with 5 normal components. However, the mixing proportion for the uniform component is negligible. According to the first step of the classification criterion, three of the normal components were designated as differential components (see Figure 2(a)).  Figure 2(b) shows the QQ-plot, which indicates a good fit of the model to the data. DIME identified around 21% (3,909) of the genes as having enriched Pol II binding quantity in OHT cell line when compared with MCF7.

#### 3.2.2. DNA Methylation

**Figure 3:**
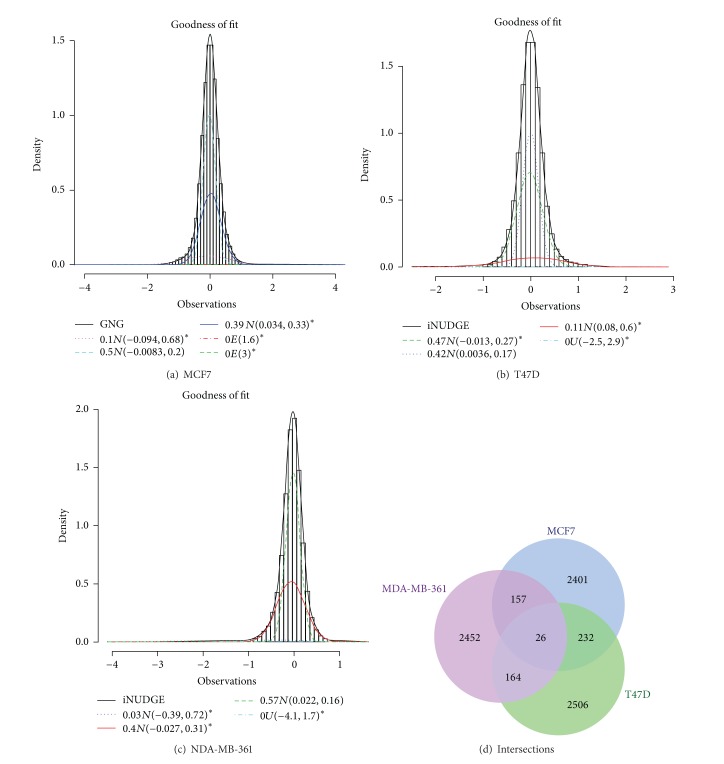
Results from fitting the weighted DIME to the DNA methylation data, where ∗ designates a differential component. ((a)–(c)) The histogram of the methylation data is superimposed by the fitted best model showing the fit of the model. Individual components of the best model are also shown to be superimposed on the histogram. (d) The Venn diagram showing the number of uniquely methylated loci in each of the three cell lines and the number of methylated loci shared between the three different cell lines.

The ensemble model was also applied to identify differentially methylated genes in three breast cancer cell lines: MCF7, T47D, and MDA-MB-361. These methylation data are from DMH microarrays that employed 2-color technique comparing a cancer cell line with a normal pooled DNA sample [12]. Lower Huber's weighting scheme was used to downweigh the log-ratios of small intensities. The overall best models selected for MCF7, T47D, and MDA-MB-361 cell lines were GNG, iNUDGE, and iNUDGE, respectively. Figures 3(a)–3(c) show that the model chosen can capture the distribution of all three cell lines well. It turns out that each estimated model has 3 normal components with negligible uniform or exponential. This indicates the need for normal component(s) to represent the differential probes, and indeed two of the three normal components were labeled as differential in each of the three cell lines. The number of probes identified to be differentially methylated is 2816, 2928, and 2799 (among around 44 k probes) for MCF7, T47D, and MDA-MB-361, respectively. The three cell lines are known to be heterogeneous and as such many uniquely methylated loci are expected. Nevertheless, since all three cell lines are hormone-receptor positive, some shared methylated loci should also be present. Indeed, the majority of the probes are uniquely methylated in each cell line but there are a few that are shared between the different cell lines (Figure 3(d)).

#### 3.2.3. Gene Expression

Dean and Raftery [16] analyzed a couple of data sets that have some known differentially expressed genes and some known similarly expressed genes, which made them valuable test data sets as it is possible to check for false positive and false negative rates.

**Figure 4:**
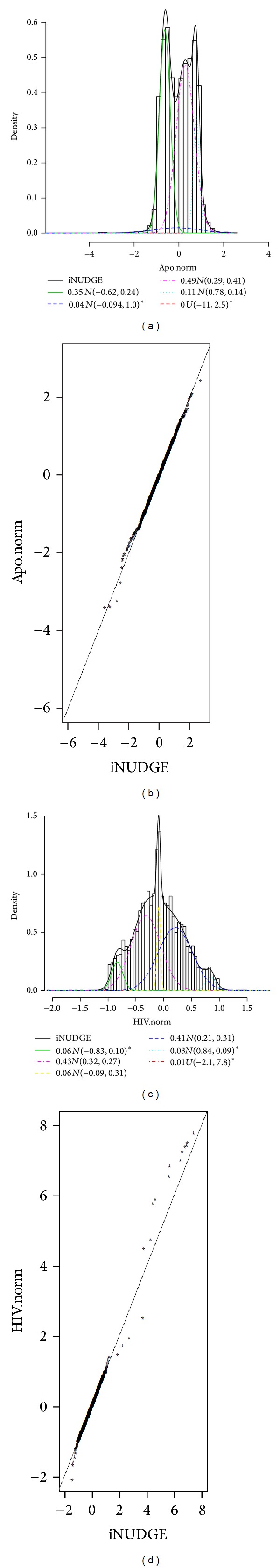
Results from fitting the DIME models to two gene expression data: ((a) and (b)) Apo AI results; ((c) and (d)) HIV results; ((a) and (c)) histograms of the normalized data superimposed by the fitted best model along with their individual components; and ((b) and (d)) QQ-plot of the fitted model versus the normalized data.

*Dataset I: Apo AI.* In this experiment, gene expression was obtained from eight normal mice and eight mice with their APO AI gene knocked out [21]. DIME yielded iNUDGE with 4 normal components as the best overall model, which indeed capture the trimodal feature of the data well (Figure 4(a)). The goodness of fit of iNUDGE is further supported by the accompanying QQ-plot (Figure 4(b)). In this model, one of normal densities was labeled as a differential component. Based on this model, 31 genes were identified as differentially expressed, which includes the 8 positive genes discussed in Dudoit et al. [21].

*Dataset III: HIV Data.* This dataset compares cDNA from CD4^+^ T cells at 1 hour after infection with HIV-1BRU and their noninfected counterparts. There were 13 genes known to be differentially expressed (HIV-1 genes, which were used as positive controls) and there were also 29 negative control genes. iNUDGE was selected once again as the best overall model for explaining the data. The density plot with 5 normal components and the QQ-plot (Figures 4(c)-4(d)) confirm the goodness of fit of the selected model. In particular, it is noted that the “spike” at the center of the distribution was quite well captured, although the QQ-plot shows disagreement between the data and the model at the right tail. There were 18 genes classified as differentially expressed, which include the 13 known positive controls. Further, none of the 29 negative control genes were included in the identified set.

## 4. Conclusions

Thanks to rapid progress and innovations in genomic research, scientists are now producing a great deal of diverse types of data in a very short period of time. These exciting developments however brought great challenges for carrying out appropriate data analysis. Existing methods designed specifically for a particular type may not lead to satisfactory results when applied to another data type. In this paper, we propose a unified differential identification approach based on an ensemble framework that is flexible enough to handle multitype data (from older/current technologies as well as potential future data). Our approach is based on classes of mixture model that have been proposed for specific data types. Here, we package these approaches into one unified framework synthesizing each of their individual advantages. In our proposed methodology, the best overall model will be selected depending on the underlying characteristics of the data and classification based on this model will be performed.

We demonstrated the applicability of our approach using both simulated and real data. We simulated data under three different underlying distributions to show the versatility of our methods for analysis for different types of data. Our results indeed show that the best model chosen by the program performed as well as or, in most cases, better than individual results from GNG, NUDGE, or iNUDGE, regardless of the underlying data types. Furthermore, it is clear from the simulation study that NUDGE is not a competitive model. The main reason for NUDGE's poor performance is due to the fact that it does not have the ability to adapt to different data types without the multiple normal components. Having multiple normal components in GNG and iNUDGE is shown to be essential to capture nondifferential elements that is not symmetrical and sometimes may even be multimodal. In our approach, labeling some normal components as differential turned out to be beneficial in that it allows the best model to capture differential data coming from any distribution, thereby increasing the flexibility of our ensemble model to capture diverse data types. Results from the analysis of three real data types all lead to reasonable goodness of fit. Further, good classification power and low error rates were obtained when applied to data with known positive and known negative controls.

Our model uses mixture of normal components with unequal variances, which can lead to a singularity problem (unboundedness in likelihood when a component variance is 0) in some cases. One suggestion to alleviate this problem to some extent is to use the BIC model selection criterion to discourage larger model (hence, less chance of having a component with observations all having the same value), which we have implemented in the package. One may also use a clustering algorithm (e.g., K-means) to provide reasonable initial starting parameters for the mixture model. In our implementation, we display a warning if potential singularity is detected. Restarting the model from different initial values and/or random seeds would also be recommended. In fact, as a good practice, our model should be run in many iterations with different starting parameters to avoid simply finding local optimum. A penalized likelihood may also be entertained to steer the variance estimates away from zero [26]. In our approach, we designate normal component as capturing differential regions based on what is commonly perceived as extreme values. Thus, the result of the classification may vary if this cut-off value is set differently.

## Figures and Tables

**Table 1:** Summary of three types of real datasets.

Data type	Description	Positive control	Negative control
ChIP-seq	Pol II comparison in MCF7 versus OHT	No	No

DNA methylation	MCF7 versus pooled normal	No	No
	T47D versus pooled normal	No	No
	MDA-MB-361 versus pooled normal	No	No

Gene expression	Apo AI knocked out versus normal mice	Yes	No
	HIV infected CD4^+^ T cell versus noninfected	Yes	Yes
